# The pathophysiology of hyperuricaemia and its possible relationship to cardiovascular disease, morbidity and mortality

**DOI:** 10.1186/1471-2369-14-164

**Published:** 2013-07-29

**Authors:** David Gustafsson, Robert Unwin

**Affiliations:** 1 Bioscience, CVMD iMED, AstraZeneca R&D Mölndal, Mölndal, Sweden; 2 University College London, UCL Centre for Nephrology, Royal Free Campus, London, UK

**Keywords:** Uric acid, Urate, Hypertension, Chronic kidney disease, Congestive heart failure, Type 2 diabetes mellitus, Metabolic syndrome, Cardiovascular events, Atherosclerosis

## Abstract

Uric acid is the end product of purine metabolism in humans. High levels are causative in gout and urolithiasis. Hyperuricaemia has also been implicated in the pathophysiology of hypertension, chronic kidney disease (CKD), congestive heart failure (CHF), the metabolic syndrome, type 2 diabetes mellitus (T2DM), and atherosclerosis, with or without cardiovascular events. This article briefly reviews uric acid metabolism and summarizes the current literature on hyperuricaemia in cardiovascular disease and related co-morbidities, and emerging treatment options.

## Review

### Physiology and pharmacology

Uric acid largely exists as urate (the ionized form, pKa is 5.8) at neutral pH. It is the end product of purine metabolism in humans. High serum levels of urate (hyperuricaemia) are causative in gout and urolithiasis, due to the formation and deposition of monosodium urate crystals. Urate is singly charged at neutral pH and at a concentration of 6.8 mg/dL (0.40 mmol/L) in human serum, crystals can form spontaneously. The solubility of urate decreases with increasing local sodium concentration, and decreasing temperature and pH [1]. The latter is an important factor in urate stone-formation in patients with acidic urine. The serum level of urate in man considered to be ‘normal’ varies among laboratories and in publications, but a range of 3.5 mg/dL (0.2 mmol/L) to 7.0 mg/dL (0.4 mmol/L) is often quoted. Serum urate is usually 0.5-1 mg/dL (0.03-0.06 mmol/L) lower in women compared with men. Serum urate levels in men have increased gradually from 3.5 mg/dl (0.2 mmol/L) in the 1920s to 6.0 mg/dL (0.35 mmol/L) in the 1970s [2]. However, no explanation for this observation has been given, but it is probably related to changes in diet, e.g., increased intake of fructose.

The serum urate level depends on dietary purines, the degradation of endogenous purines, and the renal and intestinal excretion of urate. The dominating factor contributing to hyperuricaemia is under-excretion of urate [1].

### Diet

High ingestion of purine sources (animal protein - meat and seafood - and beer) and alcohol increase the demands on purine elimination, while coffee and vitamin C reduce demand. Also, high intake of fructose increases serum urate, a relationship that has been ascribed to fructose phosphorylation in the liver with subsequent ATP depletion and regeneration [1,3].

### Purine metabolism

**Figure 1:**
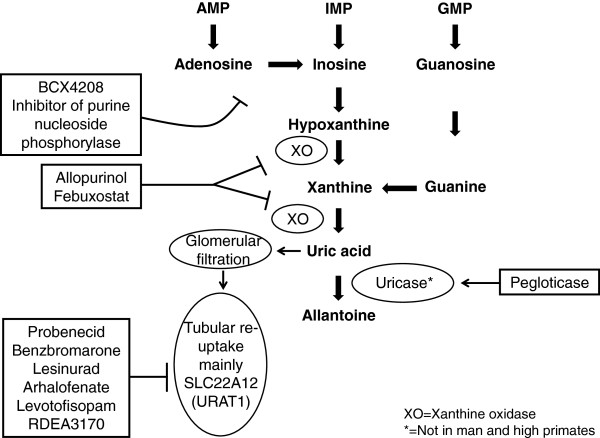
Schematic representation of uric acid formation and elimination showing the drugs that can affect both.

Increased cell turnover (e.g., haemolysis, tumour growth and large tumour necrosis) leads to increased production of adenosine, inosine and guanosine. These are degraded to hypoxantine and xanthine, which are the substrates for the widely distributed xanthine oxidase (XO) in the formation of uric acid (Figure 1). Allopurinol and febuxostat are inhibitors of XO and reduce uric acid formation. In man and some higher primates, uric acid is the end-product of purine metabolism. However, most mammals can degrade uric acid further to water-soluble allantoin by the enzyme uricase and as a result serum urate levels are about 1/10 of human values [1]. Pegloticase is a pegylated uricase that reduces urate levels by increasing its metabolism and it can be used therapeutically in man [4].

### Urate excretion

Urinary excretion accounts for two-thirds of total elimination of uric acid and the remainder is excreted in faeces. Urate is not protein bound and is freely filtered at the glomerulus, but up to 90% of filtered urate is reabsorbed (Figure 1). The main transporters responsible for tubular reabsorption are SLC22A12 (URAT1 - dominant expression is in the kidney and apical in tubular cells) and SLC2A9 (GLUT9 – widely expressed, but most likely basolateral in tubular cells), while SLC22A11 (OAT4 - apical) is less important and the evidence is weaker [5]. There also seems to be tubular secretion of urate with some evidence existing for ABCG2, SLC17A1, SLC17A3 (apical) and SLC22A6 (OAT1) and SLC22A8 (OAT3) (both basolateral) [5]. Probenecid, benzbromarone and lesinurad all inhibit URAT1, thereby reducing serum urate levels. Non-renal elimination of urate is poorly understood: a recent publication suggests that uric acid is also secreted directly into the intestinal lumen, but not via the bile [6]; studies in Caco2 cells indicate that ABCG2 is important for this route of urate elimination.

### Genetic information

Genome-wide association studies (GWAS) have implicated a number of genes associated with serum urate and gout. Hereditary renal hypouricaemia type 1 is due to loss of function mutations in URAT1 (SLC22A12) and is relatively common in Japan, and can be complicated by nephrolithiasis or exercise-induced acute renal failure [7]. URAT1 has been genetically associated with urate levels, though no genetic association has been demonstrated for URAT1 and gout [8,9]. SLC2A9 (GLUT9) is responsible for approximately 4% of the variance in serum urate levels and has an association with gout [8,9]. It is a glucose, fructose, and uric acid transporter, although its greatest affinity is for uric acid. It reabsorbs urate from the proximal renal tubule and may also be expressed in the distal nephron (and the liver and intestine); a homozygous loss-of-function mutation causes severe hereditary renal hypouricaemia type 2 [10]. ABCG2 is a renal and extra-renal urate exporter responsible for <1% of the variance of serum urate, although it has also been associated with gout [8,9]. Recently a large GWAS (>140,000 individuals) was published [11]. Of loci described previously 10 were confirmed and 18 new loci were identified. However, none of the new loci seemed to be candidates for urate transport but were instead related to glycolysis, glucose, insulin and pyruvate, indicating an importance in *de novo* purine synthesis and thereby uric acid production [11]. It was also found that alleles associated with increased serum urate concentrations were associated with increased risk of gout. Finally, the rare autosomal dominant diseases collectively known as uromodulin-associated kidney diseas (UAKD) should also be mentioned. These are characterized by hyperuricaemia, gout, and finally end stage renal disease. They are caused by mutations in the renal specific gene UMOD [12]. This suggests a coupling between urate transport and the UMOD gene product uromodulin, also known as Tamm Horsfall protein, although the details are still unclear. Uromodulin is produced by the epithelial cells of the thick ascending limb of the loop of Henle and is secreted into the urine. It is possible that uromodulin might regulate sodium transport in the thick ascending limb and that hyperuricaemia in UAKD is secondary to hypovolaemia and increased reabsorption of urate along the proximal tubule.

### Treatment of gout

Gout is the result of monosodium urate crystals in joints, which trigger the NALP3 (cryopyrin) inflammasome and release of pro-inflammatory cytokines, especially IL-1β. Gouty arthritis is often intermittent, but can be chronic, joint destructive and deforming, and persistently painful. Tophi (large crystal deposits) can form in longstanding disease. Gout affects 1–4% of adults (it is more common in the USA) and its prevalence is increasing, probably due to changes in diet, increases in CKD (gout has a higher prevalence in CKD [13]), obesity, and a longer lifespan. Subjects with serum urate between 7 and 8 mg/dL (0.40-0.48 mmol/L) have an accumulated risk of developing gout of 3%, while those above 9 mg/dL (>0.54 mmol/L) have an accumulated risk of 22%. The American College of Rheumatology guidelines recommend a serum urate target level of <6 mg/dL (<0.35 mmol/L) in all gout cases and <5 mg/dL (<0.3 mmol/L) in gout with tophi [14]. The recommendation in Japan also includes reducing hyperuricaemia, even in the absence of gout, with lifestyle guidance or drugs if >8 mg/dL (>0.48 mmol/L) and certain conditions apply [15]. Gout is costly to Society, since those affected are often absent from work [16].

Standard treatment consists of acute anti-inflammatory drugs for gouty flares, followed by the long-term urate-lowering therapy. Anti-inflammatory treatment aims at reducing the pain and swelling. Established treatments are NSAIDs and sometimes corticosteroids. Colchicine can be used as gout prophylaxis, especially when urate-lowering therapy is first introduced, but it is also used for acute gout. Drugs targeting IL-1β [4] are also effective in acute gout (e.g. rilonacept and canakinumab).

Primary urate-lowering therapy (Figure 1) is often initiated with a XO inhibitor such as allopurinol or febuxostat, but the number of patients achieving serum urate levels <6 mg/dL (<0.35 mmol/L) is in the range of 20-40% for allopurinol and 45–67% for febuxostat (Phase III data [17]), indicating the need for additional therapies. Moreover, allopurinol can have both dose-related (e.g., gastrointestinal intolerance, rashes) and idiosyncratic side effects, which can be life-threatening and may by more frequent in Asians [16,18]. Examples of non-specific uricosuric drugs are the older URAT1 inhibitors probenecid (which also inhibits OAT1 and 3), which is still available in some countries, and benzbromarone, which has largely been withdrawn because of liver toxicity. Uricosuric drugs in clinical development are mainly URAT1 inhibitors, i.e., lesinurad (Phase III), arhalofenate (Phase II and also in development for diabetes), levotofisopam (Phase II and the S-enantiomer of RS-tofisopam, an anxiolytic agent used in some countries) and RDEA3170 (Phase I) [4,19,20]. Lesinurad can achieve target serum urate levels when given with allopurinol or febuxostat in 60-100% of the patients, according to available Phase II data [16]. Use of the non-absorbable phosphate binder sevelamer can also decrease serum urate in haemodialysis patients, most likely the result of increased gastrointestinal elimination [21]. BCX4208, an inhibitor of purine nucleotide phosphorylase (an enzyme ‘higher up’ in the purine metabolic pathway), is in Phase II trial [4]. There are two pegylated uricase derivatives: pegloticase is approved for patients refractory to conventional treatments (mainly used in severe tophaceous gout) and pegadricase has been in Phase I trial and may still be in development [4]. These emerging therapies are aiming to improve efficacy and reduce side effects [4,16].

### Pathophysiology of hyperuricaemia-associated conditions

The initial trigger of the ‘inflammasome’ is from the effect of monosodium urate crystals on cells of the monocyte/macrophage lineage [22]. This leads (via the NALP3 inflammasome) to secretion of IL-1β, which then acts to recruit more inflammatory cells. The detailed mechanism underlying the secretion of IL-1β is not known, but cell damage leading to ATP release and activation of the P2X7 receptor may be involved. Potassium efflux may also be important, as well as generation of reactive oxygen species (ROS). Released IL-1β recruits other inflammatory cells and so amplifies the inflammatory reaction. The result is a burst of inflammatory mediator release. The inflammation spontaneously resolves, perhaps mediated by release of the anti-inflammatory cytokine TGF-β. The inflammasome is considered to be essential in gout and other crystalopathies, but its role in any associated pathology is less clear. It is also unclear if hyperuricaemia alone can initiate other pathological processes. What information is available, will be included with the discussion of various diseases associated with hyperuricaemia.

Multivariate analysis has been used to assess if serum urate is an independent risk factor for disease. A positive association has been found between urate levels and a number of important disorders, including hypertension, CKD, CHF, the metabolic syndrome, T2DM, endothelial cell dysfunction, cardiovascular events, and fatty liver disease. The strength of these associations will be discussed below. There are also a few intervention studies, mostly with allopurinol, but these are small and may not be representative of the effects of lowering urate by different mechanisms.

It is important to mention that urate also plays an essential function in humans. The loss of uricase in higher primates parallels the similar loss of our ability to synthesize ascorbic acid, an important anti-oxidant, leading to the suggestion that urate may partially substitute for ascorbate in humans [1,5,23]. Both uric acid and ascorbic acid are strong reducing agents (electron donors) and potent antioxidants. In humans, the major extracellular antioxidant capacity of blood comes from urate, but urate can also be pro-oxidant depending on the conditions [5,23] (see also below under the metabolic syndrome). Epidemiological data suggest that urate may be important in neuroprotection. The brain is vulnerable to oxidative stress due to its high metabolic rate and high levels of unsaturated fatty acids. Thus, increased lipid peroxidation could be one explanation for the association found between reduced serum urate levels and CNS disorders such as multiple sclerosis (MS), AML, Parkinson’s, Alzheimer’s and Huntington’s diseases. Patients with MS have significantly lower serum urate levels and there seem to be no reported cases of patients suffering from both MS and gout [24].

### Hypertension

Animal models have shown that acute elevations of serum urate (e.g., by inhibition of uricase) induce a prompt rise in blood pressure and that chronic urate elevation maintains the rise in pressure and induces irreversible vascular damage and glomerular changes, and results in a form of salt-sensitive hypertension [25,26]. The mechanisms suggested are a renin-angiotensin-aldosterone-dependent arteriolopathy, inhibition of neuronal nitric oxide synthase, and interstitial fibrosis and glomerulosclerosis with albuminuria. A meta-analysis of 11 studies showed that hyperuricaemia is associated with an increased risk of incident hypertension, independent of traditional risk factors. This risk appears more pronounced in younger individuals (with pre-hypertension) and in women [27]. In adults with essential hypertension an association with hyperuricaemia is very common.

Feig and Johnson found that about 90% of adolescent hypertension is associated with hyperuricaemia [28]. The threshold for hypertension could be as low as 5.0-5.5 mg/dL (0.30-0.33 mmol/L), clearly below the supersaturation value of 6.8 mg/dL (0.4 mmol/L). Thus, it should be independent of the formation of monosodium crystals. They also showed an effect of allopurinol, where two thirds of subjects tested normalized their blood pressure [29]. Recently, a second study published a comparison of allopurinol with probenecid, a randomized, double-blind, placebo-controlled study, in pre-hypertensive obese adolescents. The urate-lowering effect was in the same range of 6.3 to 4.1 mg/dL (0.38 to 0.24 mmol/L). Both treatments were effective, with reductions of 10 and 9 mmHg in systolic and diastolic blood pressures, respectively, suggesting that decreased urate was responsible for the effects and not decreased XO activity [30]. A systematic meta-analysis of 10 longitudinal studies (738 patients) that assessed the effect of allopurinol on blood pressure showed significant 3.3 and 1.3 mmHg decreases in systolic and diastolic blood pressures, respectively [31]. However, a recent Cochrane review only found one study fulfilling their strict criteria [29] and concluded that the data are insufficient to recommend this treatment [32]. Finally, it should be mentioned that losartan and some calcium channel blockers are uricosuric and reduce the risk of gout. Data also suggest that these agents may have a greater blood pressure-lowering effect, because their uricosuric property [33].

### Chronic kidney disease (CKD)

Animal studies with experimental hyperuricaemia (e.g., through inhibition of uricase) suggest a causative role for urate in renal disease models [34], especially if there is pre-existing renal impairment as in the 5/6 nephrectomy model [35]. In humans the situation is more complicated. A number of cross-sectional studies have found an association of urate levels with decreased eGFR or microalbuminuria, but the interpretation is difficult, because CKD can elevate urate levels and hyperuricaemia might cause or aggravate CKD. When it comes to incident CKD, most studies show an independent association with serum urate levels. However, the analysis of the progression of CKD 3–4 and its relationship to urate levels show conflicting results, most studies finding no independent association with hyperuricaemia. This could indicate that urate is more a risk factor for the onset of CKD than its progression. When it comes to kidney transplant graft loss or reduction in graft function, data are also conflicting with most studies showing no independent association with serum urate levels [34]. However, a recent review is supportive of urate as risk factor for CKD [36].

There are at least four randomized interventional studies using allopurinol in renal disease. Siu *et al.* randomized 54 patients with CKD 3-4 to allopurinol or placebo for 12 months. Allopurinol decreased systolic blood pressure (from 140 to 127 mmHg; control unchanged at 135 mmHg) and slowed CKD progression (defined as >40% rise in serum creatinine; 12 *versus* 42% of patients) [37]. Goicoechea *et al.* randomized 113 patients with eGFR <60 mL/min/1.73 m^2^ to allopurinol or usual treatment for 24 months. There was an effect on eGFR decline (defined as a decrease of >0.2 mL/min/1.73 m^2^; adjusted HR 0.53) and hs-CRP (from 4.4 to 3.0 *versus* 3.4-3.2 mg/l) favouring allopurinol treatment, and a beneficial effect on cardiovascular endpoints (7/57 *versus* 15/56), but no effect on blood pressure [38]. Momeni *et al.* randomized 40 patients with type 2 diabetes mellitus and diabetic nephropathy to allopurinol or placebo, with a reduction in proteinuria (from 1.8 to 1.0 versus 1.7 to 1.6 g per 24 h) in the allopurinol-treated group [39]. Shi *et al.* randomized 40 IgA nephropathy patients to allopurinol or usual treatment for 6 months. There was indirect evidence for a reduction in blood pressure on allopurinol (the antihypertensive drug doses were reduced in 7/9 cases with hypertension on allopurinol *versus* 0/9 in the control group), but no difference in eGFR [40]. In a *post-hoc* analysis of the RENAAL trial, losartan reduced urate levels by 0.16 mg/dL (0.01 mmol/L) from 6.7 mg/dL (0.4 mmol/L) during the first 6 months; adjustment for the urate effect indicated that 1/5 of losartan's renoprotective effect could be attributed to this reduction in urate [41]. However, it is difficult to draw any firm or generalizable conclusion for CKD, although there could be an effect of allopurinol on blood pressure and possibly an effect on eGFR.

### Congestive heart failure (CHF)

Gout is associated with CHF, subclinical measures of systolic dysfunction and mortality according to an analysis of the Framingham Offspring Study [42]. However, there also seems to be increased XO activity in the failing myocardium, perhaps due to hypoxia and apoptosis, resulting in accumulation of uric acid precursors (hypoxanthine and xanathine) and XO-induced production of ROS, causing a vicious cycle of damage [43]. There are several studies showing an association between increased serum urate levels in CHF and morbidity and mortality [43-45]. Gotsman et al. [45] in an Israeli heart failure register-based study found that treatment with allopurinol in CHF was associated with improved survival.

A group evaluating data from the Beta-Blocker Evaluation of Survival Trial took a different approach [46]. They assumed that hyperuricaemia without CKD is primarily due to increased production of uric acid from the failing heart, while hyperuricaemia in patients with CKD is in large part due to impaired renal excretion of urate. The conclusion was that hyperuricaemia is associated with a poor outcome in CHF without CKD, but not in those with CHF and CKD. This suggests that hyperuricaemia in CHF without CKD might be ascribed to increased XO activity. Although the role of XO in CHF is not clearly established, it appears that its inhibition (independent of urate-lowering) in patients with hyperuricaemia may have a beneficial effect on endothelial cell function, myocardial function and ejection fraction, while in contrast, reducing urate levels with probenecid or benzbromarone does not improve endothelial cell function or haemodynamic impairment, despite a significant decrease in serum urate level [for references see [47-50]. These data suggest that increased XO activity, rather than the serum urate level *per se*, is involved in CHF pathophysiology.

### The metabolic syndrome, T2DM and obesity

The patient with metabolic syndrome should have at least three of the following five clinical features: abdominal obesity, impaired fasting glucose, hypertriglyceridaemia, low HDL-cholesterol, and elevated blood pressure. An elevated serum urate concentration is commonly associated with the metabolic syndrome [51]; while the increase in serum urate has often been considered to be secondary, recent studies suggest that it may have an important contributory role [2]. First, elevated serum urate levels commonly precede insulin resistance, T2DM [52,53], and obesity [54], which is consistent with hyperuricaemia as a tentative causal factor; second, studies in cell culture and animal models have suggested a causative role for urate in models of the metabolic syndrome. Two mechanisms are suggested [2,23,55]: 1) hyperuricaemia-induced endothelial dysfunction, leading to reduced insulin-stimulated nitric oxide-induced vasodilatation in skeletal muscle, and as a consequence reduced glucose uptake in skeletal muscle; 2) inflammatory and oxidative changes induced by intracellular urate levels in adipocytes. For example, mice lacking XO (producing uric acid from xanthine) only have half the adipocyte mass of their wild-type littermates. A recent review [56] suggests a bidirectional and causal relationship between hyperuricaemia and hyperinsulinaemia, the former reducing nitric oxide bioavailability and the latter decreasing the renal excretion of urate. The renal clearance of urate has been found to be inversely related to insulin resistance [57], which is supported by experimental studies in healthy volunteers and hypertensive patients [58,59].

Polymorphisms in the uric acid transporter SLC2A9 (GLUT9) are associated with elevated serum urate and the risk of gout, but SLC2A9 polymorphisms are not associated with obesity or the metabolic syndrome phenotype. However, SLC2A9 exports urate out of cells [60,61], in contrast to the transporter URAT1 (SLC22A12), which mediates entry (uptake) of urate into cells. URAT1 is located on adipocytes [62,63] and URAT1 transporter gene polymorphisms in hypertensive subjects are associated with body mass index (BMI), waist circumference, HDL cholesterol, and the metabolic syndrome; - they accounted for 7% of the variation of BMI in Caucasians. However, there was no such association in African Americans [64]. In support of an involvement of adipocytes is a study in obese mice with the metabolic syndrome [65]. These mice are hyperuricaemic and lowering urate levels with allopurinol improves their pro-inflammatory phenotype in adipose tissue, with decreased macrophage infiltration and reduced insulin resistance.

A recent clinical trial studied urate-lowering with benzbromarone in patients with CHF [50]. While there was no effect on the altered haemodynamics in these CHF patients, lowering urate did improve insulin resistance. However, it cannot be excluded that it may have been a secondary pharmacological effect of benzbromarone related to its PPAR agonist activity. In a small Polish study, 28 patients with CKD were switched from a regular fructose diet to a low fructose diet for 6 weeks, and then back again. There were significant reductions in fasting serum insulin and inflammatory biomarkers, and a trend toward reductions in serum urate and blood pressure [66].

Hyperuricaemia could be a risk factor for T2DM, but a causal link remains controversial. Thus, there are studies concluding an association, no association, and even an inverse association, and have been reviewed recently by Li *et al.*[56]. Obesity is associated with reduced life-expectancy, largely because of the increased risk of cardiovascular disease. However, approximately a third of obese individuals do not develop cardiovascular disease. This group is generally referred to as the ‘metabolically healthy obese’. In a recent study, serum urate was the best predictor of ‘metabolically unhealthy obesity’ (defined as having features of the metabolic syndrome), with increased cardiovascular risk in adolescents and adults [67]. Some studies also suggest an independent association between non-alcoholic fatty liver disease (NAFLD) and hyperuricaemia [68]. Hyperuricaemia is also independently associated with the severity of steatosis and a poor response to therapy in patients with chronic hepatitis C infection [69].

It should also be mentioned that there is an increased incidence and prevalence of nephrolithiasis in patients with T2DM, and it is possible that treatment with a URAT1 inhibitor might, as a side effect, increase the risk of forming urate stones. With insulin resistance, although urinary urate levels are usually not increased (because of increased renal tubular reabsorption of urate), urinary ammonium excretion is reduced and urine pH is more acid, which increases the risk of urate crystallization [70].

### Atherosclerosis and cardiovascular events

The Framingham Heart Study reported that urate was not a risk factor for cardiovascular events, because urate was not independent of hypertension [71]. A systematic review and meta-analysis determined the risk of coronary heart disease (CHD) associated with hyperuricaemia in 26 studies with 402,997 adults. It was found that hyperuricaemia may modestly increase the risk of CHD events independently of traditional CHD risk factors. Women were found to have a more pronounced increase in risk for CHD mortality than for men [72]. A similar meta-analysis was performed for hyperuricaemia and stroke (16 studies, 238,449 adults), showing that hyperuricaemia modestly increased the risk of stroke incidence and mortality, independent of known risk factors, but without gender difference [73].

The potential relationship between hyperuricaemia and cardiovascular events could be through hypertension, but it may also involve a direct relationship due to disturbed endothelial function as a consequence of reduced nitric oxide production. Endothelial dysfunction is believed to play a key role in the early development of atherosclerosis and precedes plaque formation [74]. Endothelial-dependent flow-mediated vasodilatation of the brachial artery can assess, among other things, nitric oxide-induced vasodilatation. A recent review and meta-analysis [75] of XO inhibitors evaluated three outcome parameters and showed favourable changes in each one following XO inhibition: brachial artery flow-mediated dilatation (5 studies: XO inhibition n = 75, control n = 69) increased by 2.5% (95% CI, 0.15–4.84); forearm blood flow responses to acetylcholine infusion (5 studies: XO inhibition n = 74, control n = 74) increased by 68.8% (95% CI, 18.7–118.9; a percent change relative to the non-infused control arm); circulating markers of oxidative stress (malondialdehyde, 6 studies: XO inhibition n = 78, control n = 68) decreased by 0.56 nmol/mL (95% CI, 0.26–0.87). Three additional studies have been published following this review, two are positive [76,77] and one is negative [78]. However, it is noteworthy that short-term lowering of serum urate by intravenous uricase had no effect on forearm blood flow responses to acetylcholine and L-NMMA (n = 10 patients and n = 10 healthy subjects [79]).

## Conclusion

The present review of the available literature shows that there is an association between serum urate levels and hypertension, CKD, heart failure, the metabolic syndrome, obesity and cardiovascular events. However, as is often the case in the published literature, support is not unanimous. Understanding in the field is hampered by the difference in urate metabolism between laboratory animals and man, which makes animal studies difficult to interpret. Thus, there is limited evidence for a causal relationship. The interventional studies in man can be considered more as hypothesis-generating, since design quality, duration, and sample size are often insufficient to clarify the role of urate in cardiovascular disease. In addition, most interventional studies are with allopurinol, which is lowering urate via inhibition of XO, leading to decreased production of ROS, which may have contributed to any apparent beneficial effect. A definitive answer to the question of whether urate-lowering therapy can reduce cardiovascular morbidity and mortality will, in the end, require large interventional trials, but it is doubtful that the safety profile of allopurinol is sufficient for such large-scale studies. The recently approved and emerging novel urate-lowering agents may have a better safety profile for these much needed larger and longer-term studies. Ideally, these studies would compare cardiovascular endpoints in patients treated with placebo *versus* XO and/or URAT1 inhibition, to establish both the benefits and mechanisms of treating hyperuricaemia.

A very recent online publication has used mendelian randomization to investigate the association of plasma uric acid (*SLC2A9*) with ischaemic heart disease and hypertension (Palmer *et al*, *BMJ* 2013;347:f4262 doi: 10.1136/bmj.f4262) and concluded that there is no strong evidence for a causal association, and that the apparent link is confounded by body weight.

## Abbreviations

BMI: Body mass index; CHD: Coronary heart disease; CHF: Congestive heart failure; CKD: Chronic kidney disease; GWAS: Genome-wide association studies; MS: Multiple sclerosis; NAFLD: Non-alcoholic fatty liver, disease; ROS: Reactive oxygen species; T2DM: Type 2 diabetes mellitus; XO: Xanthine oxidase.

## Competing interests

DG is an employee of AstraZeneca and RU is consulting with AstraZeneca.

## Authors’ contributions

DG drafted the manuscript and both DG and RU revised it and approved the final manuscript. Both authors have read and approved the final manuscript.

## Pre-publication history

The pre-publication history for this paper can be accessed here:

http://www.biomedcentral.com/1471-2369/14/164/prepub
